# MoWhi2 Mediates Mitophagy to Regulate Conidiation and Pathogenesis in *Magnaporthe oryzae*

**DOI:** 10.3390/ijms23105311

**Published:** 2022-05-10

**Authors:** Shuai Meng, Jane Sadhna Jagernath, Chaoxi Luo, Huanbin Shi, Yanjun Kou

**Affiliations:** 1 State Key Lab of Rice Biology, China National Rice Research Institute, Hangzhou 311400, China; mengrice@163.com (S.M.); jane_jagernath31@hotmail.com (J.S.J.); 2 Hubei Key Lab of Plant Pathology, and College of Plant Science and Technology, Huazhong Agricultural University, Wuhan 430070, China; cxluo@mail.hzau.edu.cn

**Keywords:** mitophagy, pathogenesis, rice blast, Whi2

## Abstract

Mitophagy refers to the specific process of degrading mitochondria, which is an important physiological process to maintain the balance of mitochondrial quantity and quality in cells. At present, the mechanisms of mitophagy in pathogenic fungi remain unclear. *Magnaporthe oryzae* (Syn. *Pyricularia oryzae*), the causal agent of rice blast disease, is responsible for the most serious disease of rice. In *M. oryzae*, mitophagy occurs in the foot cells and invasive hyphae to promote conidiation and infection. In this study, fluorescent observations and immunoblot analyses showed that general stress response protein MoWhi2 is required for mitophagy in *M. oryzae*. In addition, the activation of the autophagy, pexophagy and cytoplasm-to-vacuole targeting (CVT) pathway upon nitrogen starvation was determined using the *GFP-MoATG8*, *GFP-SRL* and *MoAPE1-GFP* strains and the Δ*Mowhi2* mutant in these backgrounds. The results indicated that MoWhi2 is specifically required for mitophagy in *M. oryzae*. Further studies showed that mitophagy in the foot cells and invasive hyphae of the Δ*Mowhi2* was interrupted, leading to reduced conidiation and virulence in the Δ*Mowhi2* mutant. Taken together, we found that MoWhi2 contributes to conidiation and invasive growth by regulating mitophagy in *M. oryzae*.

## 1. Introduction

In eukaryotes, mitochondria carry out oxidative metabolism and ultimately oxidize sugars, fats and amino acids to provide energy [1]. Mitochondria are also the main source of reactive oxygen species (ROS) in cells. However, multiple signals influence mitochondria and initiate mitochondrial dysfunction. Mitochondrial dysfunction is usually multifactorial and characterized by abnormal accumulation of ROS, leading to the peroxidation of mitochondrial DNA, protein and lipid [2]. Thus, dysfunctional mitochondria may disrupt intracellular cell homeostasis and be harmful to the cells [3]. Mitophagy is a process by which dysfunctional or excess mitochondria are selectively degraded by autophagy [4]. Upon nitrogen starvation or other stresses, mitophagy receptors recognize non-essential or dysfunctional mitochondria, then recruit core autophagic proteins and encapsulate mitochondria to degradation with autophagic vesicles [5,6]. To date, the mechanism of mitophagy has been deeply studied in mammals and yeast. However, research on plant pathogenic fungi is still in the exploratory stage and requires to further study.

In addition to mitophagy receptors and the core autophagic mechanism [7,8,9,10,11], it has been suggested that mitophagy requires the general stress response protein Whi2 (Whiskey 2) in yeast [12,13]. Whi2 was initially found to play a key role in inhibiting cell proliferation by sensing extracellular nutrient unavailability in yeast [14]. Subsequent studies showed that Whi2 interacts with phosphatase Psr1 to regulate the expression of a series of stress-responsive genes through dephosphorylation of translational factor Msn2 [15]. Recent studies have shown that Whi2 inactivates the cAMP/PKA signaling pathway by targeting Ras2 (GTPase) to vacuoles [12] and negatively regulates the activity of TOR signaling complex 1 (TORC1) under limited amino acid conditions [16]. It is worth noting that according to functional analysis of the Δ*fis1* mutant with a secondary mutation in *WHI2*, loss of Whi2 function has a stronger impact on mitophagy than autophagy (macroautophagy) in yeast [13,17]. In the plant pathogens *Collectotrichum orbibulare* and *Magnaporthe oryzae*, Whi2 plays an important role in pathogenesis by regulating TOR (target of rapamycin) signaling [18,19]. Whether Whi2 specifically links to mitophagy in plant pathogenic fungi is still unclear.

Rice blast is the most devastating plant disease caused by the filamentous pathogenic fungus *M. oryzae*, resulting in a loss of rice yield, accounting for 10–30% of total rice production. At present, cultivation of rice-blast-disease-resistant varieties in combination with chemical control is the most efficient way to control this disease. In *M. oryzae*, mitophagy was first observed in foot cells. Disruption of *MoATG24*, a sorting nexin related to yeast Snx4, led to disrupted mitophagy in foot cells and a decrease in conidiation. Further research found that mitophagy of invasive hyphae was blocked in the Δ*Moatg24* mutant so that the invasive hyphae cannot spread in the host cells [20]. It is possible that MoAtg24-assisted mitophagy contributes to the transformation from biotrophy to necrotrophy in *M. oryzae* [20,21]. In addition, the mitochondrial fission machinery, including *MoDNM1*, *MoFIS1* and *MoMDV1*, is also involved in mitophagy in *M. oryzae*. MoDnm1 is not only involved in mitophagy but also in pexophagy, which mediates invasive growth in the host. MoFis1 interacts with MoDnm1 through the connection of the scaffold protein MoMdv1 to participate in mitophagy in the form of a complex and regulates appressorium formation and virulence [22]. The mitochondrial fusion gene *MoFZO1* is also required for mitophagy. Deletion of *MoFZO1* resulted in a change in mitochondria morphology and reduced invasive hyphae expansion and virulence [21]. Moreover, transcription factor MoMsn2 participates in mitophagy by regulating the expression of *MoAUH1* (3-methylglutaconyl-CoA hydratase), which affects growth, sporulation and pathogenicity [23]. The underlying mechanisms of mitophagy during development and virulence in *M. oryzae* still remain elusive and need to be further explored.

In a previous study, we found that MoWhi2 regulates appressorium formation via cAMP and MoTor signaling pathways in *M. oryzae* [19]. Here, we further demonstrate the function of MoWhi2 in mitophagy during conidiation and invasive hyphal growth. The results revealed that MoWhi2 is specifically involved in mitophagy (but not autophagy, pexophagy and the cytoplasm-to-vacuole targeting pathway) to regulate conidiation and invasive hyphae growth of *M. oryzae*.

## 2. Results

### 2.1. MoWHI2 Is Required for Mitophagy in M. oryzae

**Figure 1:**
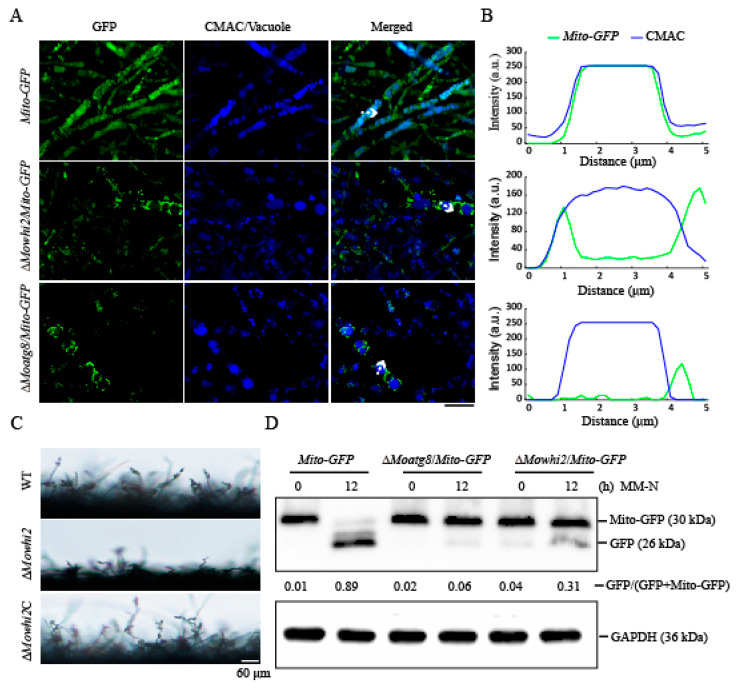
MoWhi2 is involved in mitophagy in *M. oryzae*. (**A**) The degradation of Mito-GFP was disrupted in the Δ*Mowhi2* strain. Mycelia cultured in complete media (CM) for 2 days were subjected to basal media with glycerol for 30 h and minimal media (MM) lacking a nitrogen source for 6 h. The mycelia were stained with CMAC (7-amino-4-Chloromethylcoumarin) to label vacuoles before confocal microscopic observation. Similarly to the Δ*Moatg8*/*Mito-GFP* strain, the mitochondria could not be delivered into vacuoles in the Δ*Mowhi2*/*Mito-GFP* strain. In contrast, the GFP signals were overlapped with vacuoles in the *Mito-GFP* strain. Scale bar = 10 μm. (**B**) Linescan graph analysis of the region indicated by arrow in (**A**). (**C**) Conidiophore morphology of the wild type, Δ*Mowhi2* and complemented Δ*Mowhi2*C strains. Scale bar = 60 μm. (**D**) Immunoblot analysis to detect the degradation of Mito-GFP. Equal amounts of mycelia of *Mito-GFP*, Δ*Mowhi2*/*Mito-GFP* and Δ*Moatg8*/*Mito-GFP* strains were collected at 12 h post nitrogen starvation to perform immunoblot with anti-GFP antibody. The protein GAPDH served as a loading control. The values represent the percentage of free GFP in the indicated strain analyzed by Image J. Similar results were obtained from two biological repeats.

In *M. oryzae*, mitophagy occurs in the foot cells and invasive hyphae to promote conidiation and infection [20,21]. In a previous study, we found that the deletion of *MoWHI2* not only led to abnormal appressorium formation but also highly reduced conidiation and invasive growth [19]. Thus, we speculated that MoWhi2 may participate in mitophagy as Whi2 in yeast [13]. To test this hypothesis, the *MoWHI2* deletion mutant was generated in the *Mito-GFP* background, which is a well-used strain for monitoring mitophagy in *M. oryzae* [20,21,24]. The Δ*Moatg8*/*Mito-GFP* strain, which is defective for all types of autophagy, including mitophagy, served as a negative control. The resultant mutant Δ*Mowhi2*/*Mito-GFP*, *Mito-GFP* and Δ*Moatg8*/*Mito-GFP* strains were grown in glycerol medium and transferred to nitrogen-starvation medium for 6 h to induce mitophagy. Then, the mycelia were stained with CMAC (7-amino-4-chloromethylcoumarin) dye to visualize the vacuoles and imaged with a confocal microscope. As shown in Figure 1A,B, the vacuolar localized GFP signal was evident in starved mycelia, indicating mitophagy was induced in the *Mito-GFP* strain. In contrast, similar to the negative control Δ*Moatg8*/*Mito-GFP*, the GFP signal could not be detected in the vacuoles in the Δ*Mowhi2*/*Mito-GFP* strain, suggesting that MoWhi2 is involved in mitophagy. Similarly, microscopic observations showed that few conidiophores had differentiated in Δ*Mowhi2*, revealing that the sporulation of the *MoWHI2* mutant was significantly reduced (Figure 1C). To further confirm this result, a Western blot assay was performed with anti-GFP antibody to assess the degradation of fusion protein Mito-GFP. Consistent with the fluorescent observation in the Δ*Moatg8*/*Mito-GFP* strain, deletion of *MoWHI2* resulted in a decreased degradation of Mito-GFP at indicated time points, suggesting that there are defects in mitophagy in the *MoWHI2* deletion mutant (Figure 1D). Based on these results, we concluded that *MoWHI2* is required for mitophagy in *M. oryzae*.

### 2.2. MoPsr1 Is Not Necessary for Mitophagy in M. oryzae

**Figure 2:**
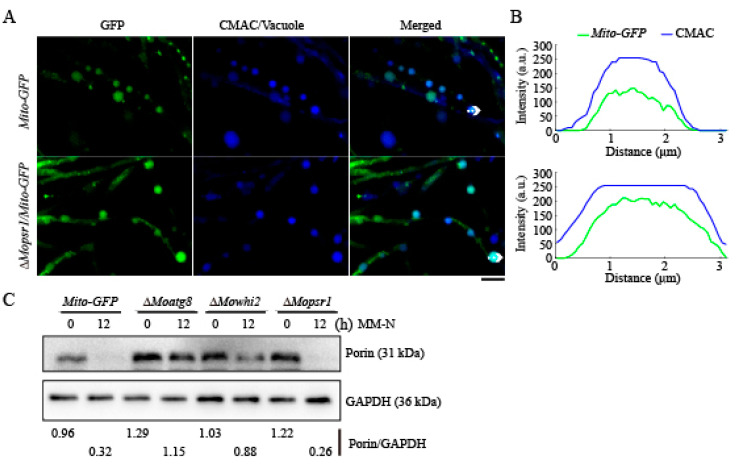
Mitophagy occurs in the Δ*Mopsr1* mutant in *M. oryzae*. (**A**) The mitochondria of the Δ*Mopsr1* strain were delivered into vacuoles upon nitrogen starvation. Similar to the *Mito-GFP* strain, the Mito-GFP-marked mitochondria were delivered into vacuoles in the Δ*Mopsr1* mutant. Scale bar = 5 μm. (**B**) Linescan graph analysis of the region indicated by arrow in (**A**). (**C**) Immunoblotting detection of the mitochondria outer membrane protein Porin. The indicated strains were cultured in liquid CM for 2 days, then shifted to BM-G (1.5% glycerol) for 30 h, followed by starvation in MM-N for 12 h. Mycelia were harvested to perform immunoblot assays with anti-Porin antibody. The values represent the percentage of Porin/GAPDH in the indicated strain analyzed by Image J. Similar results were obtained from two biological repeats.

In our previous study, we found that MoWhi2 interacts with MoPsr1, and Δ*Mopsr1* showed similar defects in conidiation and invasive growth as the Δ*Mowhi2* mutant. It is possible that MoPsr1 participates in mitophagy as MoWhi2. To test this hypothesis, we constructed a Δ*Mopsr1* mutant in the *Mito-GFP* strain. The *Mito-GFP*, Δ*Mopsr1*/*Mito-GFP* and Δ*Moatg8*/*Mito-GFP* strains were inoculated in CM medium for 2 days and then subjected to nitrogen starvation for 6 h. Confocal microscopic observation showed that the green fluorescence of *Mito-GFP* and Δ*Mopsr1*/*Mito-GFP* strains overlapped with the CMAC-stained vacuole, indicating that parts of the mitochondria are delivered into vacuoles for degradation in the Δ*Mopsr1*/*Mito-GFP* strain, as these in the *Mito-GFP* strain, upon nitrogen starvation (Figure 2A,B). These results suggest that MoPsr1 is not necessary for mitophagy. Furthermore, Western blot analyses were performed to reveal whether MoPsr1 is involved in mitophagy. The mitochondrial outer membrane protein Porin encoded by *MGG_00968* was used as a marker to detect the occurrence of mitophagy [17]. With the same protein amount, the ratios of Porin to internal reference were calculated to indicate mitochondrial degradation with nitrogen starvation treatment in the Δ*Mopsr1*/*Mito-GFP*, Δ*Moatg8*/*Mito-GFP* and Δ*Mowhi2*/*Mito-GFP* strains. The results showed that upon nitrogen starvation, part of the mitochondria in the *Mito-GFP* and Δ*Mopsr1*/*Mito-GFP* strains were degraded (Figure 2C). In contrast, the amount of Porin after induction was close to that before induction in the Δ*Moatg8*/*Mito-GFP* and Δ*Mowhi2*/*Mito-GFP* strains (Figure 2B). Taken together, MoPsr1 is not necessary for mitophagy in *M. oryzae*.

### 2.3. Deletion of MoWHI2 Did Not Affect Autophagy (Aacroautophagy) upon Nitrogen Starvation

**Figure 3:**
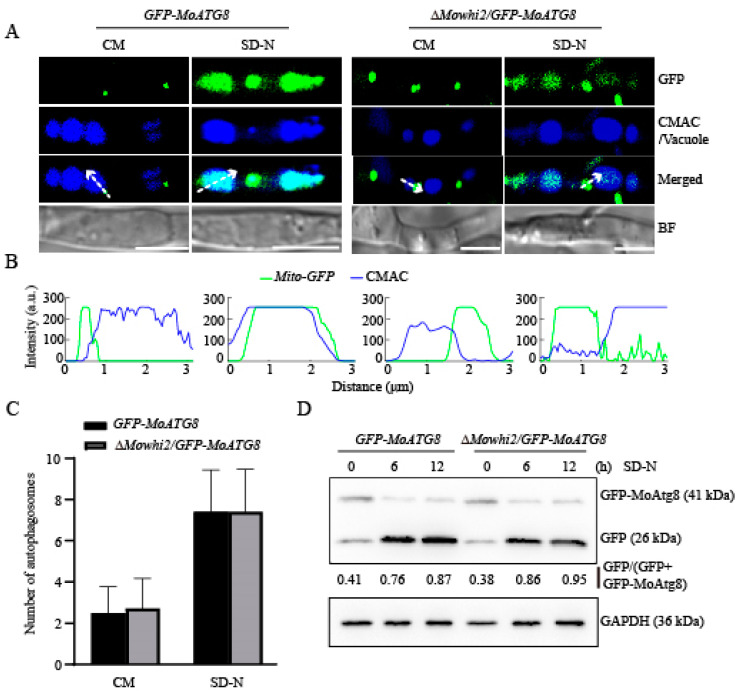
MoWhi2 is not required for autophagy upon nitrogen starvation. (**A**) Autophagy occurs in the Δ*Mowhi2* strain. Mycelia of the *GFP-MoATG8* and Δ*Mowhi2*/*GFP-MoATG8* strains cultured in CM medium were transferred to the SD-N medium for 12 h before imaging. Scale bar = 5 μm. (**B**) Linescan graph analysis of the region indicated by arrow in (**A**). (**C**) Statistics of autophagosomes in the indicated strains. Autophagosomes in more than 25 hyphal cells were counted. There were no significant differences in the numbers of autophagosomes in the hyphae of the *GFP-MoATG8* and Δ*Mowhi2*/*GFP-MoATG8* strains. (**D**) Vacuolar degradation of the GFP-MoAtg8 fusion protein was detected by immunoblotting using anti-GFP antibody. The *GFP-MoATG8* was degraded in the Δ*Mowhi2*/*GFP-MoATG8* strain as the *GFP-MoATG8* strain upon nitrogen starvation. The numbers underneath the blots are the ratios of free GFP to total GFP, indicating the level of autophagy. Similar results were obtained from three replicates.

In yeast, disruption of *WHI2* has no significant effects on autophagy [13,17]. To determine whether MoWhi2 is required for autophagy in *M. oryzae*, deletion mutants of *MoWHI2* were generated in the *GFP-MoATG8* strain, in which GFP-MoAtg8 is a commonly used marker of autophagy [25]. Under the autophagy-inductive condition, the colocalization of GFP-MoAtg8 with vacuoles was observed. As shown in the Figure 3A, the GFP fluorescence overlapped the CMAC-stained vacuoles in both *GFP-MoATG8* and Δ*Mowhi2*/*GFP-MoATG8* strains (Figure 3A,B), suggesting that autophagy could be induced when *MoWHI2* was disrupted. Furthermore, the number of autophagosomes increased in the *GFP-MoATG8* and Δ*Mowhi2*/*GFP-MoATG8* strains, but there was no significant difference between *GFP-MoATG8* and Δ*Mowhi2*/*GFP-MoATG8* in the SD-N (nitrogen starvation condition) medium (Figure 3C). In addition, the degradation of GFP-MoAtg8 fusion protein was detected by immunoblotting. The results demonstrated that the autophagy in mycelia of the Δ*Mowhi2*/*GFP-MoATG8* strain was comparable to that of the *GFP-MoATG8* stain (Figure 3D). These results suggest that MoWhi2 is not required for autophagy in response to nitrogen starvation.

### 2.4. MoWHI2 Is Not Necessary for Pexophagy and the Cytoplasm-to-Vacuole Targeting (CVT) Pathway under Nitrogen Starvation

**Figure 4:**
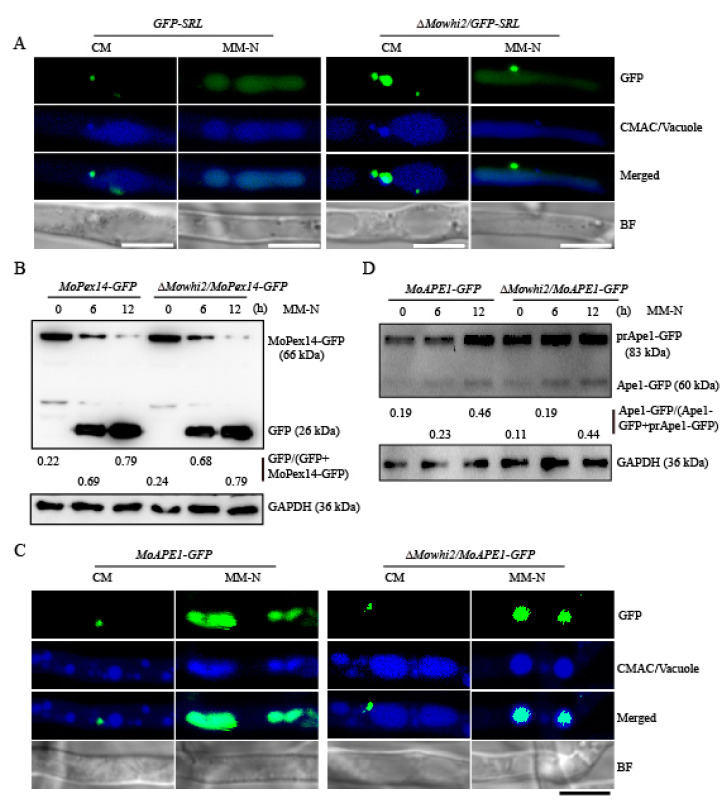
MoWhi2 is not necessary for pexophagy and the activation of the cytoplasm-to-vacuole targeting pathway in *M. oryzae*. (**A**) MoWhi2 is not necessary for pexophagy. Upon nitrogen starvation, the green fluorescence of the fusion protein GFP-SRL was overlapped with the CMAC-stained vacuoles in the Δ*Mowhi2*/*GFP-SRL* as the *GFP-SRL* strain. (**B**) Vacuolar degradation of peroxisomes was detected by immunoblotting assay. The wild-type and Δ*Mowhi2* strains expressing *MoPEX14-GFP* were cultured in CM for 2 days, then transferred to MM-N medium to induce pexophagy. The degradation dynamics of MoPex14-GFP were similar in the *MoPEX14-GFP* and Δ*Mowhi2*/*MoPEX14-GFP* strains. (**C**,**D**) MoWhi2 is not necessary for activation of the cytoplasm-to-vacuole targeting pathway in *M. oryzae*. Fluorescent imaging (**C**) and immunoblot analysis (**D**) showed the maturation of MoApe1, a marker protein of the CVT pathway, in the *MoAPE1-GFP* and Δ*Mowhi2*/*MoAPE1-GFP* strains. Similar results were obtained from three biological replicates. Scale bar = 5 μm.

Autophagy is a highly conserved process that is responsible for the recycling of cytoplasmic components by the vacuole/lysosome, including selective and non-selective autophagic processes. Pexophagy and the cytoplasm-to-vacuole targeting (CVT) pathway are involved in the selective degradation of peroxisomes and the maturation of the precursor form of aminopeptidase I or α-mannosidase, respectively [5,26,27,28]. To detect whether MoWhi2 specifically participates in selective degradation of pexophagy, deletion mutants of *MoWHI2* were generated in the *GFP-SRL* and *MoAPE1-GFP* strains. GFP-SRL is a pexosome-labelled GFP used to monitor pexosomes [29]. Moreover, the processing of the vacuolar aminopeptidase MoApe1 maturation served as an indicator of the activity of the cytoplasm-to-vacuole targeting (CVT) pathway [28,29,30]. The *GFP-SRL*, *MoAPE1-GFP*, Δ*Mowhi2*/*GFP-SRL* and Δ*Mowhi2*/*MoAPE1-GFP* strains were inoculated in CM medium for 2 days, then subjected to nitrogen starvation for 6 h. After CMAC staining, the mycelia were observed by confocal microscopy. The microscopic observation indicated that the pexophagy process occurs in both Δ*Mowhi2*/*GFP-SRL* and *GFP-SRL* strains (Figure 4A). The activity of the CVT pathway in the Δ*Mowhi2*/*MoAPE1-GFP* strain was similar to that in the *MoAPE1-GFP* strain (Figure 4C). Furthermore, the degradation rate of MoPex14-GFP, a pexophagy marker protein [31], and MoApe1-GFP was measured by immunoblot analyses. Consistent with the microscopic observation, the degradation rates of MoPex14-GFP were not significantly different in the *MoPEX14-GFP* and Δ*Mowhi2*/*MoPEX14-GFP* strains (Figure 4B), and the maturation rate of prApe1-GFP in the Δ*Mowhi2*/*MoAPE1-GFP* strain was consistent with that in the *MoAPE1-GFP* strain (Figure 4C,D). All these results suggest that MoWhi2 is not essential for pexophagy and the CVT pathway and may be specifically required for mitophagy.

### 2.5. MoWhi2 Is Necessary for Mitophagy in Foot Cells during Conidiation

**Figure 5:**
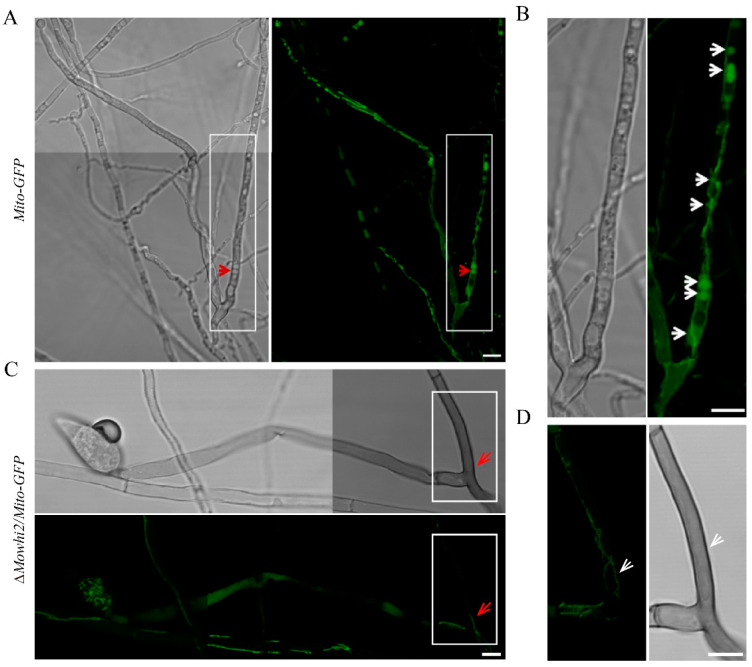
Mitophagy during conidiation in the *Mito-GFP* and Δ*Mowhi2*/*Mito-GFP* strains. Mitophagy in foot cells was affected in the Δ*Mowhi2*/*Mito-GFP* strain. Foot cells of the indicated strains were induced by culturing mycelial plugs on glass slides with a thin layer of agar medium. Pictures in (**A**–**D**) were captured at 20 hpi (hours post inoculation). Among them, panels (**B**,**D**) display magnified areas labeled by white boxes in the left panels of (**A**,**C**), respectively. The red arrows represent vacuoles and the white arrows represent mitochondria. Scale bar = 10 μm.

Mitophagy is required for conidiation in *M. oryzae* [20,21]. Given that MoWhi2 plays an important role in conidial differentiation and morphology [19], mitophagy in foot cells was determined in the *Mito-GFP* and Δ*Mowhi2*/*Mito-GFP* strains. As shown in Figure 5, the fluorescence signal of Mito-GFP was clearly presented in the vacuoles in the foot cells of the *Mito-GFP* strain, whereas the GFP signal could not be observed in the vacuoles of the Δ*Mowhi2*/*Mito-GFP* strains, suggesting that disruption of *MoWHI2* interrupts mitophagy in foot cells in *M. oryzae*. These results indicate that MoWhi2 is necessary for mitophagy in foot cells and likely contributes to conidiation.

### 2.6. MoWhi2 Is Required for Mitophagy during Invasive Growth in M. oryzae

**Figure 6:**
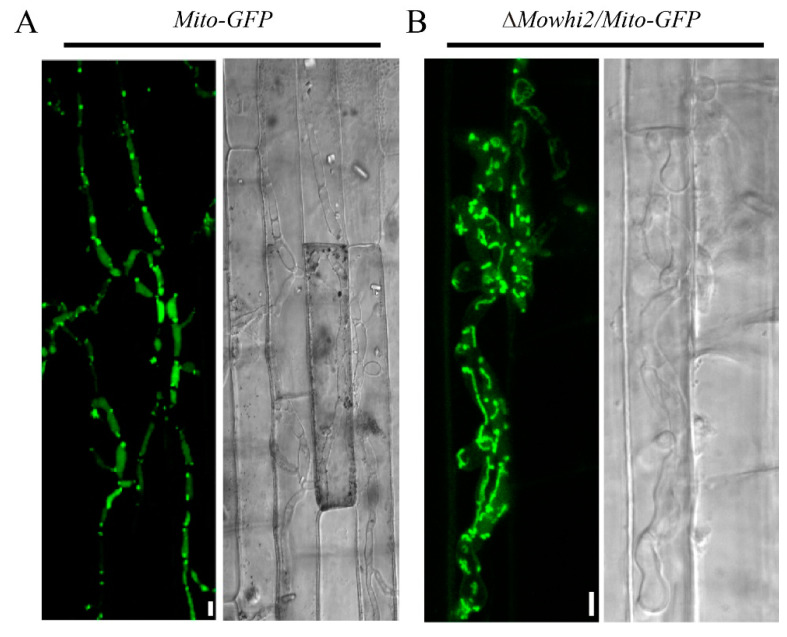
Fluorescent observation of GFP in the *Mito-GFP* (**A**) and Δ*Mowhi2*/*Mito-GFP* strains (**B**) during the invasive growth stage. Conidial suspensions of the indicated strains were inoculated on rice sheaths from 3-week-old Co39 (*Oryza sativa*) seedlings. Photographs were taken by a confocal microscope at 60 hpi.

Mitophagy is not only necessary for conidiation but also for invasive hyphae growth in *M. oryzae*. To explore the involvement of MoWhi2 in mediating mitophagy during the invasive growth stage, mitophagy in the invasive hyphae was determined in the *Mito-GFP* and Δ*Mowhi2*/*Mito-GFP* strains. In the *Mito-GFP* strain, the GFP entered into the vacuole, indicating that the mitochondria in the invasive hyphae are delivered into the vacuoles for degradation. In addition, the invasive hyphae had expanded into adjacent cells in the *Mito-GFP* strain (Figure 6A). However, the GFP signal of the Δ*Mowhi2*/*Mito-GFP* strain was outside the vacuoles, suggesting that the mitochondria could not enter the vacuoles for degradation (Figure 6B). Meanwhile, the invasive hyphae of Δ*Mowhi2*/*Mito-GFP* were mainly restricted to the first invaded cells of rice sheaths. In summary, mitophagy is impaired in the invasive hyphae of Δ*Mowhi2* in *M. oryzae*.

## 3. Discussion

In recent years, research on mitophagy has developed rapidly, especially concerning the identification of key receptors and regulatory factors [32,33], which provides a foundation for further understanding of the mechanism of this process. However, the molecular mechanism of mitophagy in plant pathogenic fungi has not been explored in detail. In this study, cell biology and biochemical analysis clearly demonstrated that MoWhi2 is specifically required for mitophagy in *M. oryzae* but not for autophagy, pexophagy and the CVT pathway. Furthermore, MoWhi2 plays important roles in mitophagy of foot cells and invasive hyphae, contributing to conidiation and virulence in *M. oryzae*.

Rice blast is a fungal disease caused by the filamentous ascomycete fungus *M. oryzae*. During the infection of *M. oryzae*, under suitable conditions, conidia germinate on the host surface to form germ tubes, which differentiate into appressoria. Turgor pressure accumulates in appressoria to penetrate the rigid rice cuticle; then, invasive hyphae grow in the host and spread to neighboring cells to form typical lesions. Conidiation is an essential step for infection, and spread of the invasive hyphae determines the formation of rice blast lesions. In *M. oryzae*, conidiation begins with the growth of a conidiophore stalk with apical extension from thick-walled vegetative cells called foot cells, which links aerial hyphae to the vegetative mycelia and ensures a supply of nutrients to the aerial hyphae [21]. Mitophagy occurs in the foot cells during conidiation in *M. oryzae*. When mitophagy in the foot cells is disrupted, conidiation is considerably reduced [20]. In addition, it was shown that mitophagy occurs in the invasive hyphae to promote invasive growth in *M. oryzae*. Knockout of the genes involved in mitophagy, such as *MoATG24*, *MoDNM1*, *MoFIS1*, *MoMDV1*, *MoFZO1* and *MoAUH1*, resulted in reduced conidiation and spreading of invasive hyphae [20,22,23]. Similarly, the Δ*Mowhi2* strain showed defects in mitophagy in the foot cells, which likely led to decreased conidia. In addition, mitophagy in invasive hyphae was disrupted in the Δ*Mowhi2* strain, leading to defective invasive growth and restricted small, dark brown spot lesions. In summary, MoWhi2 plays vital roles in mitophagy and pathogenicity by regulating mitophagy in *M. oryzae*.

Whi2 was found to be required for the induction of mitophagy in yeast more than ten years ago [13]. Similar to yeast Whi2, we found that MoWhi2 is involved in mitophagy rather than autophagy, pexophagy and the CVT pathway in *M. oryzae* (Figure 3 and Figure 4). However, not much is known about the cellular functions of Whi2 in mitophagy. In yeast, Whi2 is better known to be involved in several fundamental cellular processes. Under conditions of low extracellular nutrients, including glucose and amino acids, Whi2 inhibits cell proliferation by regulating the expression of CLN1, CLN2 and G1 cyclin; suppresses the activity of TORC1; and inactivates the cAMP/PKA signaling pathway by targeting Ras2 to vacuoles [12,16]. In addition, Whi2 interacts with phosphatases Psr1 and Psr2, as well as the transcriptional factor Msn2, to activate the expression of general stress-response genes controlled by STREs (stress-responsive elements) [15]. In *M. oryzae*, transcription factor MoMsn2 participates in mitophagy by regulating the expression level of the 3-methylglutaconyl-CoA Hydratase encoding gene *MoAUH1* [23]. In our previous study, we found that MoWhi2 also regulates appressorium formation and pathogenicity by the cAMP and MoTor signaling pathway in *M. oryzae* like ScWhi2 [19]. Based on all these results, we hypothesize that Whi2 might link cell cycle regulation, stress response, Ras-cAMP-PKA signaling pathways and TOR signaling to regulate the activity of Msn2, thus contributing to mitophagy [17].

In yeast, Whi2 and Psr1 form a complex that participates in the regulation of a series of stress responses to affect growth and development [15]. In *Colletotrichum orbiculare*, Whi2 interacts with Psr1 to promote biotrophic infection [18]. In *Podospora anserina*, Whi2 and Psr1 are necessary for sexual reproduction and nutrient-sensing signaling [34]. Moreover, the phosphatase MoPsr1, which interacts with MoWhi2, is involved in appressorium formation and pathogenicity in *M. oryzae* [19]. However, we found that MoPsr1 is not required for mitophagy. It is possible that MoWhi2 is involved in mitophagy through an MoPsr1-independent pathway. How Whi2 participates in mitophagy remains to be further studied.

In summary, we found that MoWhi2 is required for conidiation and invasive growth by regulating mitophagy in *M. oryzae*. The results deepen the understanding of the pathogenic mechanism of *M. oryzae* and provide a theoretical basis for the control of rice blast.

## 4. Materials and Methods

### 4.1. Fungal Strains and Culture Media

The *Mito-GFP*, Δ*Moatg8*/*Mito-GFP* and *GFP-SRL* strains were kindly provided by Prof. Naweed I. Naqvi of National University of Singapore (Singapore). All the *M. oryzae* strains were cultured on complete medium (CM: D-Glucose 10 g/L, Peptone 2 g/L, Casamino acid 1 g/L, Yeast extract 1 g/L, 20 × Nitrate salts 50 mL/L, 1000 × Vitamin solution 1 mL/L, 1000 × trace elements 1 mL/L, pH 6.5) under 16 h of light and 8 h of dark at 25 °C [21]. Transformants generated by *Agrobacterium tumefaciens*-mediated transformation (ATMT) were screened on basal medium (BM: yeast nitrogen base 1.6 g/L, asparagine 2.0 g/L, NH_4_NO_3_ 1.0 g/L, glucose 10 g/L, agar 20g/L, pH 6.0) with chlorimuron-ethyl (50 μg/mL). Mycelia were collected from the liquid CM media for 2 d. The strains used in this study are summarized in Supplementary Table S1.

### 4.2. Plasmid Constructions

The *MoWHI2* gene (*MGG_11241*) deletion mutant was generated using the standard one-step gene replacement strategy in the *Mito-GFP* background. Briefly, about 1 Kb of 5’ UTR and 3’ UTR regions were amplified by PCR and ligated sequentially to flanking restriction enzyme sites of the *ILV2^SUR^* sulfonylurea-resistance gene cassette in *pFGL820* (Addgene, 58221). The primers used to amplify the 5’ and 3’ UTR of the *MoWHI2* gene are listed in Supplementary Table S2. The resultant plasmid was confirmed by sequencing and subsequently transformed into the *Mito-GFP* strain by ATMT to delete the *MoWHI2* gene [35]. The Δ*Mopsr1*/*Mito-GFP*, Δ*Mopsr1*/*MoAPE1-GFP*, Δ*Mowhi2*/*GFP-MoATG8*, Δ*Mowhi2*/*MoAPE1-GFP* and Δ*Mowhi2*/*GFP-SRL* strains were constructed by a similar strategy.

### 4.3. Mitophagy, Autophagy, Pexophagy and CVT Pathway Analyses

The strains *Mito-GFP*, *GFP-MoATG8*, *GFP-SRL* and *MoAPE1-GFP* were used to monitor mitophagy, pexophagy and the CVT pathway in *M. oryzae*. For mitophagy, the *Mito-GFP* strain was first cultured in liquid CM media for 2 days, transferred to BM-G media (1.5% glycerol) for 30 h and finally transferred to MM-N media for 12 h at 28 °C [20]. To determine autophagy, pexophagy and the CVT pathway, the strains *GFP-MoATG8*, *GFP-SRL* and *MoAPE1-GFP* were cultured in liquid CM media for 2 days and then transferred to MM-N media for 12 h [28,29,36]. To stain vacuoles, the mycelia were incubated in 10 μM CellTracker™ Blue CMAC dye (7-amino-4-Chloromethylcoumarin, Molecular Probes, C2110, Carlsbad, CA, USA) for 30 min at room temperature and then washed with water before microscopic observation. Autophagosomes were counted in more than 25 hyphal cells. Linescan graph analysis was carried out by Image J software. 

To observe mitophagy in foot cells, mycelial plugs (1 cm) were cut from marginal regions of a 7-day-old colony and incubated on glass slides covered with a thin layer of 1% agar medium. The slides were placed in the dark for 2 days and cultured in the light for another 12 h at 28 °C. Foot cells were imaged with a confocal microscope after removing the mycelial plugs. To observe mitophagy in the invasive hyphae, conidial suspensions (1 × 10^5^/mL) collected from 7-day-old culture plates were inoculated on the excised rice sheaths of susceptible cultivar CO39 (*Oryza sativa*). Fluorescence of Mito-GFP in the invasive hyphae was monitored at 60 hpi.

### 4.4. Fluorescence Microscopy

A fluorescent strain with GFP signal was observed and documented by confocal fluorescent LSM700 microscopy (Zeiss, Oberkochen, Germany). GFP and CMAC were imaged with 488 nm (Em. 505–530 nm) and 405 nm (Em. 430–470 nm) laser excitation, respectively. Images were processed with Image J and arranged using Photoshop CS6 software.

### 4.5. Western Blot Assay

The mycelia (0.1 g) cultured in liquid CM for 2 days were collected at 28 °C, and the total protein solutions were extracted with lysis buffer (50 mM Tris-HCl pH 7.4, 150 mM NaCl, 1 mM EDTA, 1% Triton 100, and 1 × protein inhibitor) at 12000 rpm for 10 min at 4 °C. For detection of Porin (mitochondria outer-membrane protein), total proteins were extracted with 10% SDS solution. The resultant protein solution was resolved by 8–15% SDS polyacrylamide gel electrophoresis (SDS-PAGE) and transferred to a PVDF membrane, followed by incubation with primary antibodies anti-GFP (Huabio, ET1607-31, Hangzhou, China) or anti-porin antibody (GenScript, A01419, Nanjing, China). The protein GAPDH served as a loading control. Western blots were detected using an ECL chemiluminescent kit (Biorad GS-710, Hercules, CA, USA), and the relative intensity of blots was quantified by Image J software (version 1.37).

## Data Availability

The data presented in this study are available on request from the corresponding authors.

## Supplementary Materials

The following supporting information can be downloaded at: https://www.mdpi.com/article/10.3390/ijms23105311/s1.
