# Supplementary material for: MoWhi2 Mediates Mitophagy to Regulate Conidiation and Pathogenesis in Magnaporthe oryzae
Source: Int J Mol Sci. 2022 May 10;23(10):5311. doi: 10.3390/ijms23105311 (PMC9141721; doi:10.3390/ijms23105311)
Supplement: Supplementary file 1 [file ijms-23-05311-s001.zip › ijms-1692749-supplementary.pdf]

Table S1. ***Magnaporthe*** **strains used in this study**

Strain	Brief description	Reference
Mito-GFP	Wild-type strain with GFP-tagged mitochondria	[1]
Δ*Mowhi2*/*Mito-GFP*	*MoWHI2* deletion mutant in the *Mito-GFP* background	This study
Δ*Moatg8*/*Mito-GFP*	*MoATG8* deletion mutant in the *Mito-GFP* background	This study
GFP-MoATG8	Visualization of GFP-MoAtg8	This study
Δ*Mowhi2*/*GFP-MoATG8*	*MoWHI2* deletion mutant in the *GFP-ATG8* background	This study
GFP-SRL	Strain used for visualization of peroxisome	[2, 3]
Δ*Mowhi2*/*GFP-SRL*	*MoWHI2* deletion mutant in the *GFP-SRL* background	This study
MoPEX14-GFP	Visualization of peroxisome	This study
Δ*Mowhi2*/*MoPEX14-GFP*	*MoWHI2* deletion mutant in the *GFP-MoPEX14* background	This study
MoAPE1-GFP	Visualization of cytoplasm-to-vacuole targeting	[2, 3]
Δ*Mowhi2*/*MoAPE1-GFP*	*MoWHI2* deletion mutant in the *MoAPE1-GFP* background	This study

Table S2. Primers used in this study

Primer	Sequence (5'-3')	Application
MoWHI2-5f	TATGGAGAAACTCGAGAATTCACAAAGTGCGTAGCGAAAGTAACC	Amplifying *MoWHI2* 5' flank sequence for gene deletion
MoWHI2-5r	GACTCTAGAACTAGTGGATCCTCGTCGTCCTTGTTACTGCGC	
MoWHI2-3f	GAATTGCATGTCGACCTGCAGACGCCCAGCTGTATATGACGG	Amplifying *MoWHI2* 3' flank sequence for gene deletion
MoWHI2-3r	ACGACGGCCAGTGCCAAGCTTCTTGTCTCGGTTCCAGGTCCA	
MoWHI2-tf	CGACAGTCACTTCCACCCAA	Transformants screening
MoATG8-5F	CCCTCGAGGAATGACAGAGTCCCAGCCC	Amplifying *MoATG8* 5' flank sequence for gene deletion
MoATG8-5R	CGCGGATCCCGGCGGTTGATTGAGACTTG	
MoATG8-3F	AGATTTAGGTCGACCTGCAGTTGCCTCTGATGGACCGTTT	Amplifying *MoATG8* 3' flank sequence for gene deletion
MoATG8-3R	GGCCAGTGCCAAGCTTAGCCTTTTCAGCCTCACAGA	
MoATG8-TF	TGTGCAACTCTCAGCGTACA	Transformants screening
PATG8F	TATGGAGAAACTCGAGAATTCTGTGCAACTCTCAGCGTACAGAG	Construction of *mCherry-MoATG8*
PATG8R	ATGGCGGCGGTTGATTGAGA	
Mcatg8f	TCTCAATCAACCGCCGCCATGGTGAGCAAGGGCGAGG	
Mcatg8r	AACTTGGAGCGCTTGTACAGCTCGTCCATGCC	
atg8f	CTGTACAAGCGCTCCAAGTTCAAGGACGA	
atg8r	CTAGTGGATCCCCCGGGTACCTCACTCGACTTCCTCAAACAGGT	
MoATG8-utrF	GAGATTTAGGTCGACCTGCAGGCTTGCTCACCCGGCCGG	
MoATG8-utrR	ACGACGGCCAGTGCCAAGCTTTCCAGCCTAGCTTCTCCTCCG	
PEX14-Cf	TATGGAGAAACTCGAGAATTCTCAAGCATCCGGAGTTCCTCAAAGTGCTTGG	Construction of *GFP-MoPEX14*
PEX14-cR	CTCGCCCTTGCTCACGGTACCGCTTGACGCAGGCGGGGTTGCTGCCG	
APE1-F	TATGGAGAAACTCGAGAATTCTTGGGACCTTGCTATCGCTG	Construction of *MoAPE1-GFP*
APE1-R	CTCGCCCTTGCTCACGGTACCCTGCCACTCAGCATCAACCTC	

Reference

1. Kou Y, He Y, Qiu J, et al. Mitochondrial dynamics and mitophagy are necessary for proper invasive growth in rice blast. Mol Plant Pathol. 2019 Aug;20(8):1147-1162.
2. Zutphen T, Veenhuis M, van der Klei IJ. Pex14 is the sole component of the peroxisomal translocon that is required for pexophagy. Autophagy. 2008 Jan;4(1):63-6.
3. Li L, Wang J, Chen H, et al. Pex14/17, a filamentous fungus-specific peroxin, is required for the import of peroxisomal matrix proteins and full virulence of *Magnaporthe oryzae*. Mol Plant Pathol. 2017 Dec;18(9):1238-1252.
